# Insight into Cellular Uptake and Intracellular Trafficking of Nanoparticles

**DOI:** 10.1186/s11671-018-2728-6

**Published:** 2018-10-25

**Authors:** Parisa Foroozandeh, Azlan Abdul Aziz

**Affiliations:** 1 School of Physics, Universiti Sains Malaysia, 11800 Gelugor, Penang Malaysia; 2 Nano-Biotechnology Research and Innovation (NanoBRI), Institute for Research in Molecular Medicine (INFORMM), Universiti Sains Malaysia, 11800 Gelugor, Penang Malaysia

**Keywords:** Nanoparticles, Cellular uptake, Intracellular trafficking, Nanomedicine, Cytotoxicity

## Abstract

Nanoparticle science is rapidly changing the landscape of various scientific fields and defining new technological platforms. This is perhaps even more evident in the field of nanomedicine whereby nanoparticles have been used as a tool for the treatment and diagnosis of many diseases. However, despite the tremendous benefit conferred, common pitfalls of this technology is its potential short and long-term effects on the human body. To understand these issues, many scientific studies have been carried out. This review attempts to shed light on some of these studies and its outcomes. The topics that were examined in this review include the different possible uptake pathways of nanoparticles and intracellular trafficking routes. Additionally, the effect of physicochemical properties of nanoparticle such as size, shape, charge and surface chemistry in determining the mechanism of uptake and biological function of nanoparticles are also addressed.

## Introduction

Nanoparticles (NPs) are a subcategory of nanomaterials that are currently at the forefront of cutting-edge research in nearly every field imaginable due to its unique properties and tremendous applicability [1–4]. In a technology market research report entitled “Global NP Market Outlook 2020” by RNCOS, it was reported that the market for NPs will grow at a compound annual growth rate (CAGR) of 16% during 2015–2020. NP technology has found a unique niche in the field of biomedicine and biotechnology with its rapidly burgeoning repertoire of applications [5, 6]. For instance, NPs have been applied for drug and gene delivery [7, 8] biodetection of pathogens [9], detection of proteins [10], tissue engineering [11, 12], tumour imaging and targeting [13], tumour destruction via hyperthermia [14] and MRI contrast enhancement [15].

Owing to their small size, NPs can easily enter the cells as well as to translocate across the cells, tissues and organs. NPs are widely used in biomedical applications because they are able to pass through the biological barrier and enter the cell to exert their function. However, like a double-edged sword, the potential risks (i.e. adverse effect) of NP also arise from this capability [16, 17]. In spite of their “small” size, NPs as polar molecules are not able to diffuse through the cell membrane (CM). Since the CM is mostly permeable to small and non-polar molecules, NPs employ endocytotic pathways to enter the cells [18, 19]. The way by which NPs enter the cell is a key factor in determining their biomedical functions, biodistribution and toxicity. In nanomedicine, safe entry of NPs into the cells is a crucial step to obtain high therapeutic efficacy. Furthermore, intracellular trafficking and fate of NPs is a vital process to the success of NPs considering that these carriers are aimed to target specific sub-cellular compartment and deliver specific biomolecules such as contrast agents, genes and drugs [18, 20–22]. More importantly, the induction of cytotoxicity by NPs are determined by its entry pathway and intracellular localization. Hence, understanding cellular uptake and intracellular trafficking of NPs is crucial in designing safe and efficient nanomedicines [23].

Cellular uptake, targeting and intracellular trafficking of NPs can be optimized by tuning physicochemical properties of NP such as size, shape and surface properties [24]. Hence, knowledge of the underlying mechanisms involved in cellular uptake is crucial for assessing the fate of NPs and its toxicity. This review highlights the different possible uptake pathways of NPs and its intracellular trafficking routes. Additionally, the effect of NP’s physicochemical properties such as size, shape, charge and surface chemistry on its internalization by cells are also addressed. Understanding the physicochemical properties of NPs in relation to its cellular uptake mechanism will enable us to design functional NPs that are crucial in biomedical applications such as delivering drug payloads at the targeted site of action in a controlled manner with minimal toxic effects on the surrounding healthy tissues and organs.

## Cellular Uptake Pathways of NPs

The CM, also known as the plasma membrane, encloses the cytoplasm by detaching the intracellular from the extracellular fluid. CM is immensely important as it protects intracellular components, maintains cell homeostasis, confer structural support and retains the composition of the cell [25–29]. CM consists of phospholipids arranged in a bilayer with embedded proteins. These phospholipid bilayers, with their hydrophilic heads and hydrophobic tails, permit the entrance of small biomolecules. More specifically, the CM is a selectively permeable barrier that controls the passage of substances into the cell [30, 31]. The CM employs different mechanisms to exchange substances which are mainly divided into two categories: passive transport and active transport. Gases such as oxygen and carbon dioxide, hydrophobic molecules such as benzene and uncharged molecules such as water and ethanol diffuse across the membrane from the regions of higher to lower concentration. This kind of transport which is along the concentration gradient and occurs without assistance of energy is called passive transport. In contrast, active transport occurs against the concentration gradient by using energy which is provided by adenosine triphosphate (ATP) [32–36].

Polar or charged biomolecules that cannot pass through the hydrophobic plasma membrane are internalized by a form of active transport which is called endocytosis. In this process, the cell engulfs the materials inside the extracellular fluid by invagination of CM and buds off inside the cell, forming a membrane-bounded vesicle called an endosome [37]. Endocytosis can be basically classified into two major categories: phagocytosis and pinocytosis. Phagocytosis (cell eating) is the process of taking in debris, bacteria or other large size solutes by specialized mammalian cells called phagocytes (i.e. monocytes, macrophages and neutrophils) [38, 39].

Integral to phagocytosis is a process called opsonization by which opsonins such as immunoglobulins and complement proteins coat the target materials to trigger the phagocytes of their presence and to initialize phagocytotic activity [40]. As the phagocyte begins to ingest the target material, it will simultaneously stimulate the formation of a membrane-bound vesicle called phagosome into which the ingested materials are compartmentalized within the phagocyte. At the latter stages of this process, the phagosome will fuse with the lysosome and the materials are digested at acidic pH by the hydrolytic enzymes contained within the lysosomal lumen [41–43].

In all cell types, small particles within the range of nanometers are internalized by pinocytosis [44]. In pinocytosis, “cellular drinking” plasma membrane forms an invagination to take up a small droplet of extracellular fluid including dissolved molecules in it. Pinocytosis is not a discriminating process and it occurs in almost all the cells in a continuous manner irrespective to the needs of the cell. The grabbed substances are pinched off into small vesicles that are called pinosome which fuses with lysosomes to hydrolyze or break down the contents [45, 46]. Phagocytosis and pinocytosis can be distinguished by the size of their endocytotic vesicles; the former encompass uptake of large particles by large vesicles with the size of 250 nm, and the latter encompass uptake of fluids through small vesicles with the size in the range of a few nanometres to hundreds of nanometres [42, 47]. Pinocytosis can be subcategorized into clathrin-mediated endocytosis, caveolae-mediated endocytosis, clathrin- and caveolae-independent endocytosis and macropinocytosis [48, 49].

Clathrin-mediated endocytosis is the cellular entry mechanism to internalize specific molecules into the cells. This entry route aids cells to take in plasma membrane components and nutrients including cholesterol by low-density lipoprotein receptor and iron by transferrin receptor [50–56]. In this process, particular ligands in extracellular fluid bind to the receptors on the surface of the CM forming a ligand-receptor complex. This ligand-receptor complex moves to a specialized region of the CM which are rich in clathrin, whereby they are engulfed through the formation of clathrin-coated vesicles. Once inside the cell, clathrin coatings on the exterior of the vesicles are expelled prior to fusing with early endosomes. The cargo within early endosomes will eventually reach lysosomes via the endo-lysosomal pathway [40, 57–60]. Each type of NP is internalized by the cell via preferentially uptake pathway. For example, NPs composed of poly(lactic-co-glycolic acid), D,L-polylactide and poly(ethylene glycolco-lactide) and silica (SiO_2_)-based nanomaterials are internalized by clathrin-mediated endocytotic pathway [61]. Coumarin-based solid-lipid NPs are internalized by the cells via non-energy-dependent pathway as the structure of these NPs are similar to the CM. All the lipid-based NPs utilize the clathrin-mediated endocytosis pathway [62]. The herceptin-coated gold NPs enter the cell via receptor-mediated endocytosis by means of membrane ErbB2 receptor [63].

Caveolae-mediated endocytosis is the route of cellular entry which involves flask-shaped membrane invaginations called caveolae (little caves). Caveolae are present in endothelial cells, epithelial, adipocytes, muscle and fibroblasts cells [64–67]. The size of caveolae typically ranges from 50 to 80 nm and are composed of membrane protein caveolin-1 which confer them flask-shaped structure [68–71]. Caveolae-dependent endocytosis is involved in cell signaling and regulation of membrane proteins, lipids and fatty acids [61, 64, 67]. Once caveolae are detached from plasma membrane, they fuse with a cell compartment called caveosomes that exists at neutral pH. Caveosomes are able to bypass lysosomes and therefore protect the contents from hydrolytic enzyme and lysosomal degradation. Hence, pathogens including virus and bacteria use this entry route to prevent degradation. Since the cargo internalized into the cells by caveolin-dependent mechanism do not end up in the lysosome, this pathway is employed in nanomedicine [54, 72–74].

**Fig. 1:**
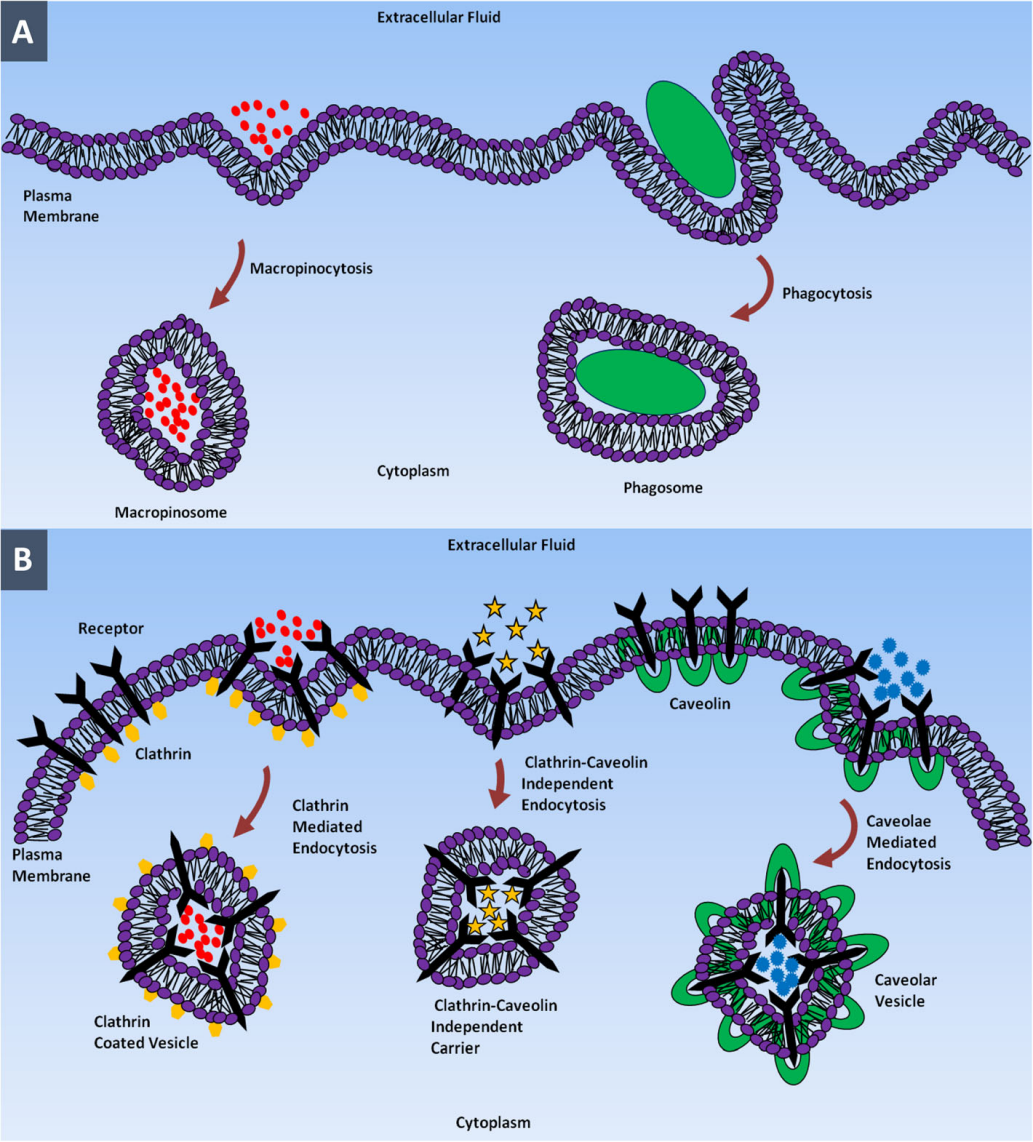
Entry of NPs into cell using different endocytotic pathways. **a** Macropinocytosis and phagocytosis. **b** Clathrin-mediated endocytosis, clathrin-caveolin independent endocytosis and caveolae-mediated endocytosis

Clathrin- and caveolae-independent endocytosis occurs in cells that are deprived of clathrin and caveolin. This pathway is utilized by growth hormones, extracellular fluid, glycosylphosphatidylinositol (GPI)-linked proteins and interleukin-2 to enter the cells. For instance, folic acid that employs clathrin- and caveolae-independent pathway to enter the cells [58, 72, 75–79] are conjugated to NPs and polymers used in drug delivery systems and as imaging agents [53, 80, 81]. Macropinocytosis is a type of pinocytosis mechanism in which cells take in high volumes of extracellular fluid by forming a large vesicle (0.5–10 μm) called macropinosomes [82–85]. Macropinocytosis is a pathway to internalize apoptotic and necrotic cells, bacteria and viruses as well as antigen presentation. This pathway can internalize micron-sized NPs which are not possible to be taken into cells by most other pathways. Macropinocytosis can occur in almost any cells except for brain microvessel endothelial cells [86–89]. NPs enter into the cell via one of these endocytotic routes as depicted in Fig. 1.

## Effect of Physicochemical Properties of NP on Cellular Uptake

**Fig. 2:**
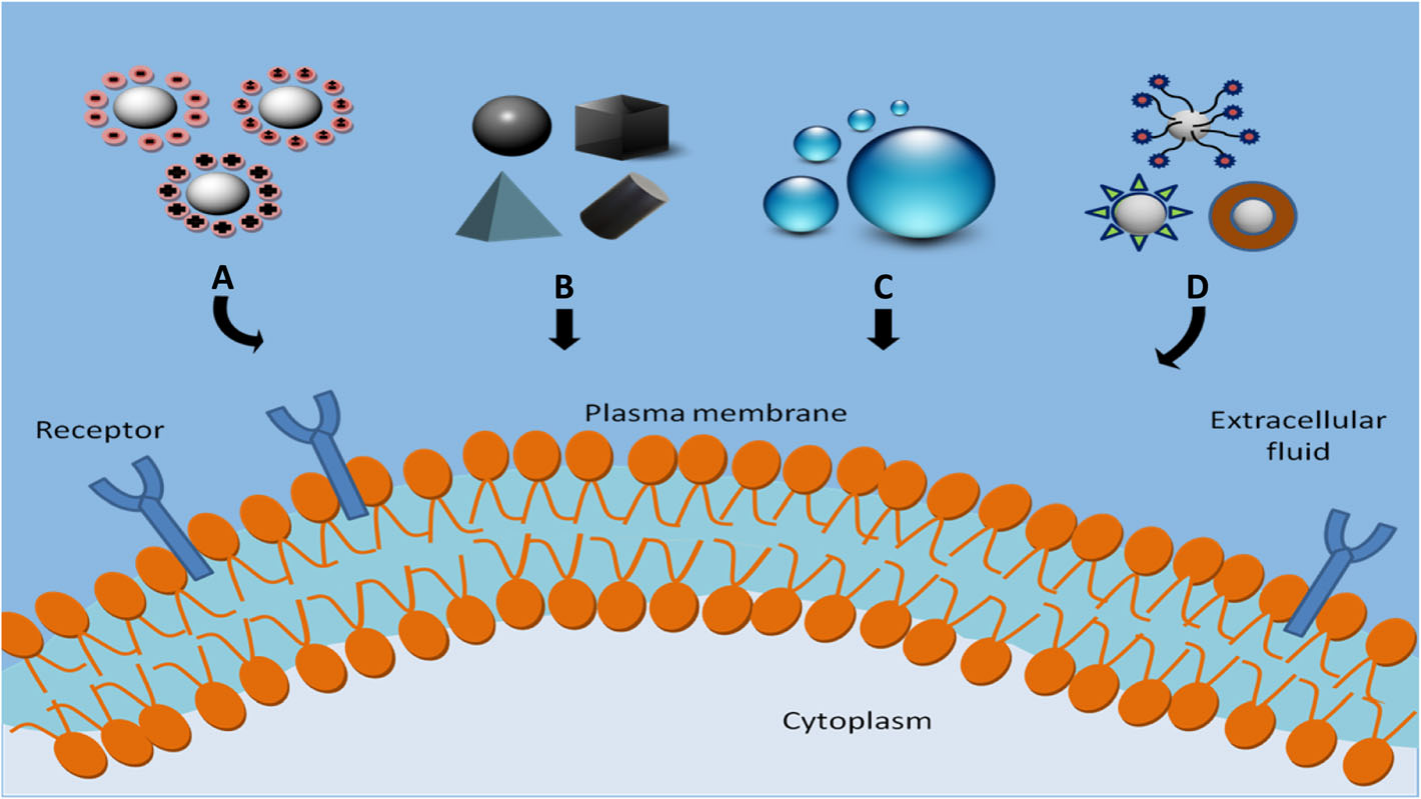
Physicochemical factors that affect cellular uptake of NP. **a** Surface charge, **b** shape, **c** size and **d** surface chemistry

Studying the effect of physicochemical properties of NPs such as size, shape, surface charge, surface hydrophobicity/hydrophilicity and surface functionalization on cellular uptake is crucial as these parameters directly affect the uptake level, endocytotic route as well as cytotoxicity of NPs. [90, 91]. Physicochemical factors that affect the cellular uptake of NPs are illustrated in Fig. 2. In the following section, the impact of these parameters on cell–NP interactions are discussed.

### Effect of Size

Size of NP is a key factor in determining the efficiency of cellular uptake [92] as well as its toxic potential on living cells [24]. Moreover, the size of NP was found to play a major role in determining the uptake pathway as well. Small NPs with sizes ranging from a few to several hundred nanometers enter the cells via pino- or macropinocytosis. NPs in the size range of 250 nm to 3 μm have been shown to have an optimal in vitro phagocytosis, whereas NPs with the size range of 120–150 nm are internalized via clathrin- or caveolin-mediated endocytosis, and the maximum size of NPs employing this pathway was reported to be of 200 nm [47, 93]. In the caveolae-mediated pathway, the size of caveolae hinders the uptake of larger NPs [16, 17]. A particular type of NP may utilize multiple endocytic pathways depending on its size.

Several studies have indicated that for cellular uptake of NPs, there is an optimum size of 50 nm at which NPs are internalized more efficiently and has a higher uptake rate. NP uptake was shown to decrease for smaller particles (about 15–30 nm) or larger particles (about 70–240 nm) [94–99]. Additionally, NPs ranging in the size of 30–50 nm interacts efficiently with CM receptors and is subsequently internalized via receptor-mediated endocytosis [97]. In drug delivery application of NPs, the main concern is to prevent the NPs from being eliminated by the reticuloendothelial system and to prolong its circulation time in the blood, thus enhancing the bioavailability at the target. In this regard, increasing the size of NPs will lead to an increase in the clearance rate [100–105]. Therefore, understanding the role of NP size in cellular uptake is crucial to design effective and safe NPs for medical applications.

Though different studies have investigated the relationship between size of NP and uptake pathways, the revealed results have always been inconsistent [93, 106–109]. These contradictions can be related to the complexity of controlling other parameters of NP during the process of controlling size. In addition to that, sizes of NPs measured after synthesis may undergo changes during the in vitro and in vivo studies due to agglomeration and aggregation which in turn could affect the cellular internalization pathways [110, 111]. The impact of particle size on cellular uptake pathway in non-phagocytic B16 cells was investigated by employing different sizes of fluorescent latex beads in the range of 50–1000 nm [93]. The results have demonstrated that the internalization mechanism of these beads relies significantly on the particle size. In particular, beads with sizes of 200 nm or less were taken up by clathrin-coated pits whereas larger beads were internalized by caveolae-mediated endocytosis. Lai and co-workers [16] have found that small polymeric NPs with sizes less than 25 nm employs a new mechanism to reach the perinuclear region of the cells via non-degradative vesicle outside the endo/lysosomal pathway. This pathway is non-clathrin and non-caveolae-mediated and cholesterol-independent.

The uptake of gold (Au) NPs of different sizes (2 to 100 nm) conjugated with Herceptin-AuNPs by SK-BR-3 cells was shown to be size dependent. The highest cellular internalization was observed for NPs in the size ranges of 25–50 nm [63]. In this entry route, the size of NP was found to be the determinant in the binding and activation of membrane receptors and the eventual expression of the proteins. The effect of variation in the size and shape of colloidal AuNPs on the intracellular uptake was assessed [112]. AuNPs of 14-, 50- and 74-nm size with spherical and rod shape were incubated with HeLa cells. It was found that the NP uptake strongly depends on its size and shape and those particles with 50 nm size showed the highest uptake rate. Moreover, the uptake of spherical AuNPs was 500% more than rod-shaped NPs of similar size. Shan et al. [113] investigated the size-dependent force of endocytosing AuNPs with diameters of 4, 12 and 17 nm by HeLa cells. The results revealed that both the uptake and unbinding force values increase by the size of AuNPs. The uptake of SiO_2_ NPs of different sizes (50, 100 and 300 nm) by A549 cells (lung epithelial cells) has studied by means of combination of flow cytometry, fluorescence and electron microscopies. These researchers had shown that the uptake of SiO_2_ NPs has decreased by size [114].

### Effect of Shape

In addition to size, the shape of the NP also plays a pivotal role in the uptake pathway as well as trafficking of NPs. Chithrani et al. [112] studied the effect of the shape of colloidal AuNPs on the uptake of HeLa cells. The result revealed that spherical AuNPs had five-fold higher uptake than rod-shaped AuNPs. In another work, same researchers investigated the uptake level of spherical and rod-shaped transferrin-coated AuNP on three different cell lines; STO cells, HeLa cells and SNB19 cells [94]. They observed that spherical AuNPs were internalized by all the cell lines at a higher rate than rod-shaped AuNPs.

In order to establish the effect of shape in vivo, Geng and coworkers [115] employed filomicelles to evaluate the differences in transport and trafficking of flexible filaments with spheres in rodents. The results revealed that filomicelles remained in the circulation about ten times more than spherical counterparts. Moreover, the sphere filomicelles are internalized by the cells more readily than longer filaments. Gratton and co-workers [106] demonstrated the effect of the shape of monodisperse hydrogel particles on uptake into HeLa cells. They have found that rod-like-shaped NPs had the highest internalization rates compared to spheres, cylinders and cubes. In another study, the impact of the shape of NPs on cell uptake was investigated by employing disc-shaped, spherical and rod-shaped polystyrene (PS) NPs on Caco-2 cells. The result demonstrated that the rod and disc-shaped NPs were internalized twofold higher than spherical NPs. They concluded that NP-mediated drug delivery can be advanced by considering the shape of NPs [116].

Xu and co-workers [117] studied the impact of shape on cellular uptake by preparing layered double hydroxide (LDH) NPs with fluorescein isothiocyanate (FITC) in different morphology such as hexagonal sheets (50–150 nm laterally wide and 10–20 nm thick) and rods (30–60 nm wide and 100–200 nm long). All morphologies were taken up via clathrin-mediated endocytosis. LDH-FITC nanosphere were retained in the cytoplasm, whereas LDH-FITC nanorods were moved towards the nucleus by microtubules. Dasgupta et al. applied [118] a simulation to probe the role of the shape of NPs on cellular uptake. They have simulated membrane wrapping of the nanorod- and nanocube-shaped NPs. For rod-like particles, they found stable endocytotic states with small and high wrapping fraction; increment in aspect ratio was undesirable for complete wrapping. Nangia and Sureshkumar [119] have computerized the effect of shape on the translocation rate of NPs by applying advanced molecular dynamics simulation techniques. A major revelation of the study is the significant variation in the translocation rate of cone-, cube-, rod-, rice-, pyramid- and sphere-shaped NPs.

### Effect of Surface Charge

Another critical factor which influences cellular uptake of NPs is surface charge. In the recent decade, nano surface modification has been employed to engineer the surface charge of NPs to be either cationic or anionic [92]. The negatively charged CM enhances the uptake of positively charged NPs. In particular, positively charged NPs have higher internalization than neutral and negatively charged NPs [47, 120]. However, the uptake of positively charged NPs may disrupt the integrity of CM and lead to an increase in toxicity [121, 122]. In general, positively charged NPs induce cell death [123, 124]. Interestingly, neutrally charged NPs will lower the cellular uptake as compared to negatively charged NPs [110, 125–127]. Moreover, the internalization of negatively charged NPs leads to gelation of membranes, while positively charged NPs cause fluidity in the CM [128, 129]. In addition to the uptake rate of NP, surface charges also affect the uptake mechanisms. More specifically, positively charged NPs are mainly internalized by the cell via macropinocytosis whereas clathrin-/caveolae-independent endocytosis is the mechanism for the uptake of negatively charged NP [130]. Cellular uptake pathways vary when the surface of the AuNPs is coated by organic molecules. For instance, plain AuNPs which are positively charged, are internalized via macropinocytosis and clathrin and caveolin-mediated endocytosis, while negatively charged polyethylene glycol (PEG) coated AuNPs are mainly internalized via caveolin- and/or clathrin-mediated endocytosis [131].

Li and Gu [132] studied the interaction of charged and neutral NPs with CM by means of molecular dynamics simulations. It was found that charged NPs had better adhesion to the CMs compared to neutral NPs. Moreover, by increasing the charge density of NPs, they can be fully wrapped by the membrane. Another research group employed molecular dynamics simulation to investigate the interactions of cationic and anionic AuNPs with CMs. The results have revealed that disruption to the CM due to AuNPs penetration increases as the charge density of AuNPs are enhanced [133]. These findings suggest a way of controlling the interactions between cells and AuNP by manipulating the surface charge densities of AuNPs to optimize its uptake while minimizing cytotoxicity which are essential characteristics for any NPs that are being considered for biomedical applications.

Li and Malmstadt [134] studied the interaction of positively and negatively charged PS-NPs with biological membrane. The result showed that the strong electrostatic interaction between cationic NPs and the phosphate groups of the membrane led to enhance NP–membrane binding and membrane surface tension which in turn result in the formation of pores. The uptake rate of positively charged AuNPs into SK-BR-3 cells was reported fivefold higher than negatively charged AuNPs. These researchers have also explored that positively charged AuNPs were internalized by non-endocytosis pathways while negatively charged AuNPs were taken up by cells via endocytosis pathways [135].

Hauck et al. [107] probed the uptake of gold nanorods (AuNRs) with a size range of 18 to 40 nm and surface charges in the range of + 37 mV to − 69 mV by HeLa cells. The results indicated that for all concentration of AuNRs, the highest internalization into HeLa cells was with the surface charges of + 37 mV and the lowest internalization at − 69 mV. Huhn and coworkers [136] assessed charge-dependent interactions of colloidal AuNPs with different cell lines such as 3T3 fibroblast cells, murine C17.2 neural progenitor cells and human umbilical vein endothelial cells. The result showed that for all the cell lines cationic AuNPs had higher uptake than the anionic counterpart. They concluded that the cell uptake is highly dependent on the sign of charge. Moreover, the cytotoxicity study indicated that as a consequence of higher uptake for positively charged NPs, they show higher toxicity than negatively charged one.

### Effect of Hydrophobicity

Hydrophobicity of NP is a determinant factor in their interaction with the CM [92, 137]. Several studies demonstrated the impact of hydrophobicity of NPs on their interactions with the CM. Li et al. [138] studied the effect of hydrophobicity/hydrophilicity of NPs on the interaction with CM by employing molecular dynamics simulations. The results have revealed that hydrophobic NPs created inclusion in the CM while hydrophilic NPs was found to adsorb onto the CM. In another research, simulation approach was applied to investigate the effect of hydrophobicity on NP-cell interaction. It was observed that hydrophilic NPs were wrapped, while hydrophobic NPs were embedded within the inner hydrophobic core of the bilayers by directly penetrating into the membrane [139].

QDNPs interactions with mixed lipid/polymer membranes were assessed by changing the hydrophobicity surface of NPs. It was observed that hydrophobic NPs have located within the polymer domains in a mixed lipid/polymer monolayer of the membranes, whereas hydrophilic QDNPs adsorbed onto the monolayers and spread throughout, indicating higher effect on the molecule packing at the air/water interface [140]. Incorporation of functionalized AuNPs with mixed hydrophobic and hydrophilic ligands into liposome walls was studied. The result demonstrated that hydrophobic ligands interact with the hydrophobic core of the bilayer, while hydrophilic ligands interact with the aqueous solution [141].

### Effect of Surface Modification

In biomedical applications of NPs, surface chemical modification of NP is a critical step utilized to decreases toxicity, increase stability and to control and modulate cellular internalization of NPs, hence their biological fate [142]. Surface functionalization of NPs predominantly comprises of PEG, the negative carboxyl (–COOH) group, neutral functional groups like hydroxyl (–OH) groups, and the positive amine (–NH2) group. The increment in the amount of (–NH2) lead to an enhanced positive surface charge, and hence raise the uptake of NPs into cells [143–146]. Similarly, –COOH functional groups increase the negative charge of NPs and accordingly enhance its uptake [144].

Tao et al. [147] have designed polydopamine functionalized NP-aptamer bioconjugate for tumour targeting. They have reported that the functionalized NPs have better targeting efficacy compared to non-functionalized NPs, indicating higher cellular uptake rates for functionalized NPs which translates into enhanced therapeutic effect. In another research, folic acid-functionalized NPs demonstrated higher efficacy in the targeting of cervical cancer cells than non-functionalized NPs [148]. The impact of surface coating on toxicity and cellular uptake of AuNPs were studied by Qiu and co-workers [90]. They have revealed that surface coating is a key factor in determining the cellular uptake rate since poly (diallyldimethyl ammonium chloride)-coated AuNRs showed a higher efficiency in internalization by the cells.

The differences in the cellular uptake of pristine polystyrene (PS-NPs) and amino-functionalized polystyrene NPs were investigated by Jiang and co-workers [149]. The results have demonstrated that amino-functionalized polystyrene NPs have a higher uptake rate than PS-NPs, and the former were internalized mainly via clathrin-mediated pathway and the latter via clathrin-independent endocytosis. This remarkable difference highlights the key role of surface chemical modification in cellular interactions with NPs. Surface-modified fullerene, C_60_*(*C*(*COOH*)*_2_*)*_2_ NPs were internalized by the cells predominantly via endocytosis in a time-, temperature- and energy-dependent manner. Clathrin-mediated endocytosis was found to be the preferred pathway for the internalization of C_60_*(*C*(*COOH*)*_2_*)*_2_ NPs [150].

### Effect of Elasticity

The elasticity of NPs plays is an intrinsic factor in influencing its internalization by cells. The elasticity of NPs can be explained by its resistance to changes when forces are applied on it. Stiffness, hardness and rigidity are some of the terms that are synonymous in describing the elasticity of NPs. A measurement index that is being used to gauge the elasticity of NPs is Young’s modulus and the unit of measurement is Pascal (Pa). Based on this measurement, a higher Young’s modulus value denotes higher NPs elasticity and vice versa. Examples of the analytical devices or instruments that are used to measure this value on NPs are atomic force microscope, rheometer and nanoindenter. NPs that have higher elastic values are called hard NPs and examples of these are gold NPs, quantum dots and magnetic NPs. NPs that have lower elastic values are called soft NPs and examples of these are hydrogels, liposomes and biodegradable polymers.

Numerous studies that have focused on this parameter of NP with respect to cellular uptake have reported on the preference of cells to internalize stiffer NPs more efficiently compared to softer NPs [151, 152]. Evidently, this observation is attributed to lesser overall energy expenditure by membranes in wrapping stiffer NPs compared to softer NPs even though the deformational energy required to wrap the NPs varies throughout the internalization process. Furthermore, computational modelling of membrane wrapping of NPs with varying elasticity conducted using coarse-grained molecular dynamics (CGMD) simulation concurs with the experimental observation regarding deformational energy changes involved in internalizing stiff and soft NPs [153]. However, there are also other studies that have reported on softer NPs being internalized more efficiently than stiffer NPs [154, 155] and intermediate elastic NPs internalized more efficiently compared to either stiff or soft NPs [156]. Hence, tuning the elasticity of NPs for better cellular internalization could be a valuable tool in biomedical applications such as drug delivery. A potential application was demonstrated by Guo and coworkers, whereby accumulation of nanolipogels in tumour cells were enhanced primarily by controlling this parameter of NP [157].

## Intracellular Trafficking of NPs

In the previous sections, different possible uptake pathways of NPs and the parameters that affect the efficacy of uptake has been discussed. Following uptake, the next crucial matter is the intracellular trafficking of NPs which determines its final destination within cellular compartments, its cytotoxicity and its therapeutic efficacy [158, 159]. After NPs are internalized by the cells, they will first encounter membrane-bound intracellular vesicles called early endosomes. Endosomes formed at the plasma membrane are categorized into three types; early endosomes, late endosomes and recycling endosomes [106, 160–163].

Early endosome ferries the cargo to the desired cellular destination. Part of the cargo is recycled to the plasma membrane via recycling endosomes. Early endosomes transform into late endosomes via maturation and differentiation process. The late endosomes will then integrate with lysosomes to form endolysosomal vesicles and hydrolytic enzymes contained within these vesicles degrade the trapped NPs [18, 164–166]. However, some NPs are able to escape this pathway and are released into the cytoplasm therefore bypassing the lysosomal degradation process [167–169]. Another intracellular degradation pathway which plays important role in the intracellular fate of NPs is an intracellular process called autophagy [170–172]. In this process, cytoplasmic contents will be surrounded by autophagosome and delivered to the lysosome to be broken down and recycled [173]. In addition, aggregated proteins and dysfunctional organelles are degraded by autophagy to maintain cellular homeostasis. It is necessary to consider this pathway since recent studies demonstrated that several NPs are capable of inducing autophagy [174–178]**.**

The intracellular trafficking of Tat peptide-conjugated quantum dots (Tat-QDs) in live cells was studied by Ruan and co-workers [179]. Dynamic confocal imaging showed that Tat-QDs interacted with negatively charged CMs leading to its internalization by macropinocytosis. The QD containing vesicles were observed to be actively transported by molecular motors towards the perinuclear region known as the microtubule-organizing center (MTOC). Tat-QDs bind to cellular membrane structures such as filopodia and vesicle shedding results in releasing QD-containing vesicles from the tips of filopodia.

The uptake and intracellular fate of fluorescent carboxylated polystyrene particles (20 nm and 200 nm in diameter) were evaluated by applying it on hepatocyte [180]. It was found that the particles were internalized by hepatocytes in size, time and serum-dependent manner. The fate of the particles was studied and they were not observed in early endosomes or lysosomes, but only in the mitochondria of the hepatocyte. Particles accumulated inside bile canaliculi show that NPs can be eliminated within bile. A study on the uptake and intracellular fate of silver NPs into human mesenchymal stem cells demonstrated that they agglomerate in the perinuclear region [181]. It was observed by using fluorescent probes that particles are contained within endo-lysosomal structures but not in the cell nucleus, endoplasmic reticulum or Golgi complex. Confocal imaging of FITC conjugated titania nanotubes in mouse neural stem cells revealed that they have crossed the karyotheca entering the cell nucleus [182]. Single-walled carbon nanotubes were observed to enter the cytoplasm and localize in the cell nucleus leading to cell mortality [183]. Translocation of AuNRs towards the nucleus has also been reported [184].

## Conclusions

The application of NPs in the modern world is growing at an exponential rate as the scientific enterprise is looking for novel ways to address current problems. NPs can be found as active ingredients in many formulations intended for human consumption, from cosmetics to processed foods. As its application increases in consumer products, so does human exposure to NPs. Hence, more research should be carried out to understand its potential hazards to humans and other living beings. In this review, we have looked at the current knowledge on the effects of NPs at a cellular level. Some of the topics discussed include cellular pathways of NPs and the influences of physiochemical properties of NPs on the uptake rate and uptake mechanism.

## Abbreviations

ATP: Adenosine triphosphate
CAGR: Compound annual growth rate
CM: Cell membrane
FITC: Fluorescein isothiocyanate
GPI: Glycosylphosphatidylinositol
LDH: Layered double hydroxide
MTOC: Microtubule-organizing center
NP: Nanoparticle
PEG: Polyethylene glycol
